# Supplementary material for: Dissecting the effect of single- and co-infection of TB and COVID-19 pathogens on the sputum microbiome
Source: Microbiol Spectr. 2026 Mar 3;14(4):e02220-25. doi: 10.1128/spectrum.02220-25 (PMC13055300; doi:10.1128/spectrum.02220-25)
Supplement: Supplemental materials — Supplemental text, figures, and tables. [file spectrum.02220-25-s0001.pdf]

Supplementary Information

for the manuscript: Dissecting the effect of single- and co- infection of TB and COVID-19 pathogens on the sputum microbiome

Contents

Supplementary Text 2

Supplementary Tables 9

Supplementary Figures 17

Supplementary Files 24

Supplementary Text

Definitions of key measures

This section gives the definitions of the different measures/metrics used in our study.

Relative abundance (also denoted as proportion p): Given a sample, the relative abundance is calculated by dividing the count x_i of a particular taxon i in the sample by the total count of all s taxa within the sample.

$$p_i = \frac{x_i}{\sum_{j=1}^{s} x_j}$$

CLR transformation: Given a sample, the CLR transformation is applied by taking the logarithm of the ratio of the count x_i of a particular taxon i in the sample to the geometric mean of all s taxa counts within the sample.

$$CLR_i = log\frac{x_i}{\left(\prod_{j=1}^{s} x_j\right)^{\frac{1}{s}}}$$

The same transformation can also be applied to a vector of non-count data for a sample, such as the vector of pathway activity scores of a sample estimated by PICRUSt2.

Alpha (α) diversity: Different α-diversity measures were used to assess the distribution of taxa within a community [1]. We have used the following α-diversity measures in this study.

1. Shannon (diversity) index: This index quantifies the uncertainty in the distribution of taxa within a sample and is given by:

   $$\text{Shannon index} = -\sum_{i=1}^{s} p_i \ln p_i$$

   Here, s is the total number of taxa and p_i is the relative abundance of taxa i in a sample as calculated above.
2. Observed richness/taxa: This measure represents the total number of distinct taxa observed in a sample (i.e., the number of taxa i for which $p_i > 0$).

3. Simpson (diversity) index: This measure gives the probability that two randomly selected individuals from a community will have the same taxa. Its formula is given by:

$$\text{Simpson index} = 1 - \sum_{i=1}^{s} p_i^2$$

Bray-Curtis dissimilarity: It is a statistical measure used to quantify the dissimilarity in taxa composition between two samples from the same group.

$$\text{Bray-Curtis dissimilarity(A, B)} = \frac{\sum_{i=1}^{s} |a_i - b_i|}{\sum_{i=1}^{s} (a_i + b_i)}$$

Here, a_i and b_i are the abundances (read counts) of taxa i in samples A and B respectively.

ICC: It is a statistical measure used to assess the degree of agreement or consistency between two or more different measurements or raters and is calculated based on the estimates of population variances. We have used the ICC absolute agreement formula which states that "Each target is rated by a different judge and the judges are selected at random" [2]. In our analysis, repeated samples represent the target, and Shannon diversity serves as the rating. The number of repeats correspond to the number of judges, representing sequencing performed across different batches.

$$ICC(1,1) = \rho_{1,1} = \frac{\sigma_r^2}{\sigma_r^2 + \sigma_w^2} \tag{1}$$

Here, σ_r^2 denotes the variance between targets, and σ_w^2 the variance within targets (across sequencing batches).

Dispersion analysis: It is used to assess the degree of variability (or spread) of microbial composition within or between data groups. We have used betadisper function of vegan package in R to evaluate the homogeneity of group dispersions between each pair of disease groups [3, 4]. This function calculates the distances of each sample from its group centroid

in an ordination space based on the Bray–Curtis dissimilarity matrix, providing a measure of variability within the group. ANOVA was then used to test significant differences in variabilities between the groups.

Network and interaction analysis

We compared the species-species association network of TB group with that of the TBCOVID group to identify species pairs that are differentially associated between the two groups. The networks were constructed using the SpiecEasi method, which considers associations as conditional dependence relationships between taxa after accounting for all other taxa in the community. On visually examining the two networks (Fig. S6), we observed a few shared associations such as *Rothia mucilaginosa-Granulicatella adiacens* and *Dialister invisus-Dialister pneumosimtes*, and several associations that were present in the TB but not in the TBCOVID network. But a statistical test of differential association (Fisher's z-test at significance level 0.05 after multiple testing correction) did not reveal any significant rewiring of the network between the groups. We also used the interaction analysis framework [5] developed by our lab to quantify the interaction effects in the presence of co-infections and no significant interactions were detected at the genus and species levels.

Assessment of generalizability across independent cohorts

Our cohort of TB, COVID, TBCOVID, and Control participants is not geographically diverse, as all participants were recruited from a single region (Chennai) in India. To evaluate the robustness of our findings on differential taxa between Control vs. disease groups, we applied our pipeline to the following two independent cohorts:

1. A COVID cohort comprising 44 healthy and 52 COVID samples from China [6].
2. A TB cohort comprising 16 healthy and 50 untreated TB samples from Karnataka, India [7].

As no TBCOVID cohorts were available, the analyses were limited to comparisons between Control vs. individual disease groups. The same pipeline used in our study was

applied to the above cohorts to identify differentially abundant taxa at both genus and species levels. The taxa identified as differentially abundant in our cohort, referred to as "hits" were tested for replication in these external cohorts. As only *Alloscardovia* was enriched with LFC $\geq$1.5 (log fold change, as determined by LinDA using base 2) in the Control vs. COVID comparison within our cohort, we also tested differentially abundant taxa identified in the Control vs. TBCOVID groups within our cohort for replication in the external COVID cohort (at both genus and species levels). The results from these analyses are summarized below:

- In the replication testing of COVID-related hits from our study (from Control vs. COVID and Control vs. TBCOVID comparisons), we found that 5 genera identified as differentially abundant hits in our Control vs. TBCOVID analysis were also significantly differentially abundant in the external cohort (Control vs. COVID) (see Table S4). At the species level, two hits were found to be significant in the external cohort (see Table S5).
- In the replication testing of Control vs. TB hits from our study in the external cohort, consistent abundance patterns were observed for *Mycobacterium tuberculosis*, *Solobacterium moorei* and *Prevotella oris* (see Tables S6 and S7).

We would like to note that the external TB cohort included only 16 control samples, which may partly explain the fewer overlapping differentially abundant taxa between cohorts. Nevertheless, the consistency of these patterns across datasets highlights the robustness of our findings.

References

[1] **Finotello F, Mastrorilli E, Di Camillo B**. 2018. Measuring the diversity of the human microbiota with targeted next-generation sequencing. *Briefings in bioinformatics* 19 (4):679–692.

[2] **Revelle W, Revelle M**. 2015. Package 'psych'. *The comprehensive R archive network* 337 (338):161–165.

[3] **Anderson MJ, Ellingsen KE, McArdle BH**. 2006. Multivariate dispersion as a measure of beta diversity. *Ecology letters* 9 (6):683–693.

[4] **Dixon P**. 2003. VEGAN, a package of R functions for community ecology. *Journal of Vegetation Science* 14 (6):927–930.

[5] **Subramanian N, Philip P, Rajamanickam A, Kumar N, Babu S, Narayanan M**. 2023. Delineating markers of disease-disease interaction: a systematic methodology and its application to multiple diabetes-helminth cohorts. *bioRxiv* p 2023–12.

[6] **Wu Y, Cheng X, Jiang G, Tang H, Ming S, Tang L, Lu J, Guo C, Shan H, Huang X**. 2021. Altered oral and gut microbiota and its association with SARS-CoV-2 viral load in COVID-19 patients during hospitalization. *npj Biofilms and Microbiomes* 7 (1):61.

[7] **Hazra D, Chawla K, Sintchenko V, Magazine R, Martinez E, Pandey A, et al.**. 2024. The impact of anti-tuberculosis treatment on respiratory tract microbiome in pulmonary tuberculosis. *Microbes and Infection* p 105432.

[8] **Pollard, TJ and Johnson, AEW and Raffa, JD and Mark RG**. 2018. tableone: An open source Python package for producing summary statistics for research papers. *JAMIA Open* 1 (1):26–31.

[9] **Khan AA, Khan Z**. 2020. COVID-2019-associated overexpressed Prevotella proteins mediated host–pathogen interactions and their role in coronavirus outbreak. *Bioinformatics* 36 (13):4065–4069.

[10] **Wong KK, Wu BG, Chung M, Li Q, Darawshy F, Tsay JCJ, Holub M, Barnett CR, Kwok B, Kugler MC, et al.**. 2025. Microbial contribution to metabolic niche formation varies across the respiratory tract. *Cell Host & Microbe* .

[11] **Segal L, Clemente J, Tsay J, Koralov S, Keller B, Wu B, et al.**. 2016. Enrichment of the lung microbiome with oral taxa is associated with lung inflammation of a Th17 phenotype. *Nature Microbiology* 1 (5):1–11.

[12] **Larsen JM**. 2017. The immune response to Prevotella bacteria in chronic inflammatory disease. *Immunology* 151 (4):363–374.

[13] **Charles, Angel and Hernandez, Denise and Bucci, Madelyn and Wallet, Shannon and Maile, Robert**. 2025. 515 Prevotella Melaninogenica-derived Metabolites Induce Immune Reprogramming of Human THP-1 Macrophages. *Journal of Burn Care & Research* 46 (Supplement_1):S113–S113.

[14] **Lu S, Zhou Y, Hu Y, Wang J, Li H, Lin Y, Wang D, Xian J, Zhao S, Ma J, et al.**. 2023. Metatranscriptomic analysis revealed Prevotella as a potential biomarker of oropharyngeal microbiomes in SARS-CoV-2 infection. *Frontiers in cellular and infection microbiology* 13:1161763.

[15] **Haran JP, Bradley E, Zeamer AL, Cincotta L, Salive MC, Dutta P, Mutaawe S, Anya O, Meza-Segura M, Moormann AM, et al.**. 2021. Inflammation-type dysbiosis of the oral microbiome associates with the duration of COVID-19 symptoms and long COVID. *Jci Insight* 6 (20):e152346.

[16] **Bourumeau W, Tremblay K, Jourdan G, Girard C, Laprise C**. 2023. Bacterial Biomarkers of the Oropharyngeal and Oral Cavity during SARS-CoV-2 Infection. *Microorganisms* 11 (11):2703.

[17] **Qin M, Ding W, Qin L, Liang R, Guo Y, Zhao Y, Xu H, Wen Y, Pang Y, Li L**. 2025. Dysbiosis associated with enhanced microbial mobility across the respiratory tract in pulmonary tuberculosis patients. *BMC microbiology* 25 (1):499.

[18] **Cheung MK, Lam WY, Fung WYW, Law PTW, Au CH, Nong W, Kam KM, Kwan HS, Tsui SKW**. 2013. Sputum microbiota in tuberculosis as revealed by 16S rRNA pyrosequencing. *PloS one* 8 (1):e54574.

[19] **Gudowska-Sawczuk M, Mroczko B**. 2022. The role of nuclear factor kappa B (NF-κB) in development and treatment of COVID-19. *International journal of molecular sciences* 23 (9):5283.

[20] **Alteri CJ, Mobley HL**. 2012. Escherichia coli physiology and metabolism dictates adaptation to diverse host microenvironments. *Current opinion in microbiology* 15 (1):3–9.

[21] **Rowlett VW, Mallampalli VK, Karlstaedt A, Dowhan W, Taegtmeyer H, Margolin W, Vitrac H**. 2017. Impact of membrane phospholipid alterations in Escherichia coli on

cellular function and bacterial stress adaptation. *Journal of bacteriology* 199 (13):10–1128.

[22] **Cut TG, Mavrea A, Cumpanas AA, Novacescu D, Oancea CI, Bratosin F, Marinescu AR, Laza R, Mocanu A, Pescariu AS, et al.**. 2023. A retrospective assessment of sputum samples and antimicrobial resistance in COVID-19 patients. *Pathogens* 12 (4):620.

[23] **Liu HH, Yaron D, Piraino AS, Kapelusznik L**. 2021. Bacterial and fungal growth in sputum cultures from 165 COVID-19 pneumonia patients requiring intubation: evidence for antimicrobial resistance development and analysis of risk factors. *Annals of clinical microbiology and antimicrobials* 20 (1):69.

[24] **Ma S, Zhang F, Zhou F, Li H, Ge W, Gan R, et al.**. 2021. Metagenomic analysis reveals oropharyngeal microbiota alterations in patients with COVID-19. *Signal Transduction and Targeted Therapy* 6 (1):191.

[25] **Lamoureux C, Guilloux CA, Courteboeuf E, Gouriou S, Beauruelle C, Héry-Arnaud G**. 2021. Prevotella melaninogenica, a sentinel species of antibiotic resistance in cystic fibrosis respiratory niche? *Microorganisms* 9 (6):1275.

[26] **Poppleton DI, Duchateau M, Hourdel V, Matondo M, Flechsler J, Klingl A, Beloin C, Gribaldo S**. 2017. Outer membrane proteome of Veillonella parvula: a diderm firmicute of the human microbiome. *Frontiers in microbiology* 8:1215.

[27] **Tenaillon O, Skurnik D, Picard B, Denamur E**. 2010. The population genetics of commensal Escherichia coli. *Nature reviews microbiology* 8 (3):207–217.

Supplementary Tables

Table. S1 **Summary of the clinical characteristics of TB and TBCOVID groups generated using the tableone [8] package in Python:** Here, BPL indicates Below Poverty Line. IP refers to the Intensive Phase of TB treatment (initial phase, typically 2 months), while CP denotes the Continuation Phase (follow-up phase, typically 4–6 months). Pos-smokeless indicates positive for smokeless tobacco use. RIF, INH, FQ, SLI, and SLI (eis) indicate resistance to Rifampicin, Isoniazid, Fluoroquinolones, Second-Line Injectable drugs (SLI), and Kanamycin due to enhanced intracellular survival (eis) gene-associated resistance, respectively and these resistance categories were determined through targeted drug susceptibility testing (DST).

P-values were calculated using default tests in tableone, viz., Chi-squared test for categorical variables and ANOVA for continuous variables.

		Grouped by COVID Result			
		Overall	Negative	Positive	p-value
n		48	24	24	
Age (in years), mean (SD)		52.2 (11.4)	52.5 (10.4)	52.0 (12.5)	0.861
	range (min - max)	25 - 80	36 - 75	25 - 80	
Gender, n (%)	female	7 (14.6)	3 (12.5)	4 (16.7)	1.000
	male	41 (85.4)	21 (87.5)	20 (83.3)	
Socio Economic Status, n (%)	BPL	38 (79.2)	20 (83.3)	18 (75.0)	0.544
	Unknown	10 (20.8)	4 (16.7)	6 (25.0)	
New or Previously treated, n (%)	New	48 (100.0)	24 (100.0)	24 (100.0)	1.000
Weight (in kilograms), mean (SD)		46.2 (10.3)	45.5 (10.8)	46.9 (9.9)	0.658
	range (min - max)	27 - 69	32 - 69	27 - 65	
Height (in centimeters), mean (SD)		158.8 (6.7)	158.8 (6.9)	158.7 (6.8)	0.970
	range (min - max)	144 - 176	148 - 176	144 - 168	
Treatment regimen, n (%)	Normal	48 (100.0)	24 (100.0)	24 (100.0)	1.000
Treatment Phase, n (%)	CP	8 (16.7)	3 (12.5)	5 (20.8)	0.701
	IP	40 (83.3)	21 (87.5)	19 (79.2)	
Treatment outcome, n (%)	Cured	32 (66.7)	16 (66.7)	16 (66.7)	0.347
	Lost to follow up	6 (12.5)	4 (16.7)	2 (8.3)	
	On Treatment	5 (10.4)	1 (4.2)	4 (16.7)	
	Treatment Complete	4 (8.3)	3 (12.5)	1 (4.2)	
	Died	1 (2.1)		1 (4.2)	
HIV status, n (%)	Non Reactive / Negative	48 (100.0)	24 (100.0)	24 (100.0)	1.000
Diabetes, n (%)	Diabetic	27 (56.2)	13 (54.2)	14 (58.3)	1.000
	Non-diabetic	21 (43.8)	11 (45.8)	10 (41.7)	
Tobacco-Smoking, n (%)	No	31 (64.6)	15 (62.5)	16 (66.7)	0.345
	Unknown	1 (2.1)	1 (4.2)		
	Yes	14 (29.2)	8 (33.3)	6 (25.0)	
	Pos-smokeless	2 (4.2)		2 (8.3)	
Alcohol, n (%)	No	25 (52.1)	11 (45.8)	14 (58.3)	0.563
	Yes	23 (47.9)	13 (54.2)	10 (41.7)	
Rifampicin, n (%)	Not Detected	47 (97.9)	24 (100.0)	23 (95.8)	1.000
	-	1 (2.1)		1 (4.2)	
Smear, n (%)	1+positive	19 (39.6)	8 (33.3)	11 (45.8)	0.297
	2+positive	15 (31.2)	10 (41.7)	5 (20.8)	
	3+positive	14 (29.2)	6 (25.0)	8 (33.3)	
RIF, n (%)	Not detected	48 (100.0)	24 (100.0)	24 (100.0)	1.000
INH(InhA), n (%)	Not detected	48 (100.0)	24 (100.0)	24 (100.0)	1.000
INH(KatG), n (%)	Not detected	47 (97.9)	24 (100.0)	23 (95.8)	1.000
	Detected	1 (2.1)		1 (4.2)	
FQ class resistance, n (%)	Not detected	1 (100.0)		1 (100.0)	1.000
SLI, n (%)	Not detected	1 (100.0)		1 (100.0)	1.000
SLI (eis), n (%)	Not detected	1 (100.0)		1 (100.0)	1.000
COVID Result, n (%)	Negative	24 (50.0)	24 (100.0)		< 0.001
	Positive	24 (50.0)		24 (100.0)	

Table. S2 **Sample distribution summary:** This table gives the batch-wise distribution of samples in each group.

Group/Batch	Batch 1	Batch 2	Batch 3	Batch 4	Batch 5	Total	Number of samples excluding repeat samples
**TB**	4	2	12	8	1	**27**	**24**
**COVID**	2	4	1	1	4	**12**	**10**
**TBCOVID**	4	2	11	10	0	**27**	**24**
**Control**	2	4	1	1	18	**26**	**24**
**Total**	**12**	**12**	**25**	**20**	**23**	**92**	**82**

Table. S3. **Summary of differentially abundant taxa and their possible mechanisms in TB–COVID co-infection:** The summary is populated based on extensive literature review.

**Differentially enriched species**	**Associated pathways**	**Hypothesized mechanisms**	**Clinical outcomes**	**Reported in COVID-only studies**	**Reported in TB-only studies**
**Prevotella melaninogenica*	Pentose phosphate, gondoate biosynthesis, 6-hydroxymethyl -dihydropterin diphosphate biosynthesis, NAD biosynthesis pathways	Overexpression of *Prevotella* proteins may promote NF-κB pathway activation [9]; increased generation of metabolites [10]; increased Th17 inflammatory response [11]	Increased inflammation leading to TB progression or COVID-19 severity [9, 12]; can lead to acute lung injury and pneumonia [13]	[14, 15, 16]	[17, 18]
**Veillonella parvula*	L-arginine synthesis related pathways	Can play a role in activation of NF-κB signaling pathway	Can promote inflammation [19]	[15, 16]	[18]
***Escherichia coli*	Phospholipid and membrane synthesis, amino acid synthesis, and carbon metabolic pathways	Generation of ammonia due to catabolism of amino acids [20]; alteration of membrane lipid composition [21]	Can promote increased virulence and antimicrobial resistance [20]	[22, 23]	
+*Capnocytophaga gingivalis*	Gondoate biosynthesis pathway	—	—	[24]	

*These taxa are part of the normal lung microbiota, but disease conditions can disrupt their symbiotic relationship with the host and other microbes [25, 26].
**A commensal member of the gut microbiota, but its presence in lungs can result in dysbiosis [27].
+Fewer than 10 reads support the associated pathways.

Table. S4 **Replication testing in the COVID cohort (at genus level):** Of the 7 genera identified as differentially abundant in our Control vs. COVID/TBCOVID analysis, 5 were detected in the external COVID cohort (i.e., taxa present in more than 5% of samples with read counts greater than 5). As mentioned above in Section **Assessment of generalizability across independent cohorts**, this analysis also includes the differentially abundant taxa identified in our TBCOVID comparison. Here, ↑ denotes the genera enriched in TBCOVID compared to Control group in our analysis, while * indicates that similar abundance pattern was also observed in the new cohort (as determined using ANCOMBC or LinDA at FDR < 0.2).

Taxa	ANCOMBC p-value	ANCOMBC adjusted p-value	ANCOMBC LFC ($\log_e$)	LinDA p-value	LinDA adjusted p-value	LinDA LFC ($\log_2$)
Anaeroglobus **↑***	0.012060	0.015075	0.559143	0.002635	0.004392	0.877886
Campylobacter **↑***	0.000003	0.000016	1.592515	0.000005	0.000012	2.187545
Kingella **↑***	0.000011	0.000027	1.497985	0.000005	0.000012	2.397098
Megasphaera **↑***	0.024774	0.024774	1.115968	0.031445	0.031445	1.558926
Prevotella **↑***	0.001551	0.002584	1.089536	0.003679	0.004599	1.368602

Table. S5 **Replication testing in the COVID cohort (at species level):** Of the 11 species identified as differentially abundant in our Control vs. COVID/TBCOVID analysis, 7 were detected in the external COVID cohort (i.e., taxa present in more than 5% of samples with read counts greater than 5). As mentioned above in Section **Assessment of generalizability across independent cohorts**, this analysis also includes the differentially abundant taxa identified in our TBCOVID comparison. Here, the red arrows correspond to the Control vs. COVID comparison, while the black arrows correspond to the Control vs. TBCOVID comparison. Furthermore, ↑ denotes enrichment and ↓ denotes depletion of species in the COVID/TBCOVID group relative to Control group in our analysis. An asterisk (*) indicates that similar abundance pattern was also observed in the new cohort (as determined using ANCOMBC or LinDA at FDR < 0.2).

Taxa	ANCOMBC p-value	ANCOMBC adjusted p-value	ANCOMBC LFC ($\log_e$)	LinDA p-value	LinDA adjusted p-value	LinDA LFC ($\log_2$)
Prevotella melaninogenica ↑*	0.002003	0.007012	1.566364	0.001503	0.003507	2.332826
Saccharibacteria_(TM7)_[G-1] bacterium_HMT_352 ↓	0.003609	0.008422	1.326483	0.000944	0.003304	2.427422
Neisseria oralis ↓*	6.42E-05	0.00045	-1.78755	0.000538	0.003304	-2.47098
Cardiobacterium hominis ↓	0.955944	0.963759	-0.01883	0.233609	0.272544	0.648046
Oribacterium asaccharolyticum ↓	0.328025	0.574044	0.351442	0.009257	0.01296	1.427953
Dialister pneumosintes ↑	0.682986	0.95618	-0.13613	0.351684	0.351684	0.486351
Anaeroglobus geminatus ↓↑*	0.963759	0.963759	-0.01037	0.005507	0.009637	0.889059

Table. S6 **Replication testing in the TB cohort (at genus level):** Of the 17 genera identified as differentially abundant in our Control vs. TB analysis, 9 were detected in the external TB cohort (i.e., taxa present in more than 5% of samples with read counts greater than 5). Here, ↑ denotes genera enriched and ↓ denotes genera depleted in the TB group relative to Control group in our analysis. An asterisk (*) indicates that similar abundance pattern was also observed in the new cohort (as determined using ANCOMBC or LinDA at FDR < 0.2).

Taxa	ANCOMBC p-value	ANCOMBC adjusted p-value	ANCOMBC LFC ($\log_e$)	LinDA p-value	LinDA adjusted p-value	LinDA LFC ($\log_2$)
Alloprevotella ↑	0.010723	0.024126	-2.23405	0.019656	0.058967	-2.35978
Granulicatella ↓	0.4872	0.5481	0.472999	0.035677	0.080274	2.316147
Lautropia ↓	0.397599	0.511199	0.769166	0.071244	0.128239	2.509357
Megasphaera ↑	0.336993	0.505489	-0.82465	0.856853	0.856853	-0.1672
Mycobacterium ↑*	0	0	1.774877	0.000715	0.006439	4.088864
Porphyromonas ↑	0.056995	0.10259	-1.58901	0.50668	0.651446	-0.92225
Prevotella ↑	0.008446	0.024126	-1.41406	0.72549	0.816177	-0.39562
Solobacterium ↓*	0.010504	0.024126	-1.7041	0.203962	0.305943	-1.49206
Streptococcus ↓	0.728394	0.728394	0.151828	0.019615	0.058967	1.938709

Table. S7 **Replication testing in the TB cohort (at species level):** Of the 23 species identified as differentially abundant in our Control vs. TB analysis, 10 were detected in the external TB cohort (i.e., taxa present in more than 5% of samples with read counts greater than 5). Here, ↑ denotes species enriched and ↓ denotes species depleted in the TB group relative to Control group in our analysis. An asterisk (*) indicates that similar abundance pattern was also observed in the new cohort (as determined using ANCOMBC or LinDA at FDR < 0.2).

Taxa	ANCOMBC p-value	ANCOMBC adjusted p-value	ANCOMBC LFC ($\log_e$)	LinDA p-value	LinDA adjusted p-value	LinDA LFC ($\log_2$)
Prevotella melaninogenica ↑	0.035839	0.071678	-2.12422	0.005904	0.013126	-4.40767
Solobacterium moorei ↓*	0.016042	0.040106	-1.64105	0.004456	0.013126	-3.93232
Lautropia mirabilis ↓	0.34747	0.425705	0.832215	0.922727	0.922727	0.142321
Prevotella pallens ↑	0.011595	0.038649	-2.36942	0.000223	0.002228	-5.07397
Cloacibacterium normanense ↓	0.560567	0.560567	0.460773	0.644011	0.805014	-0.5924
Megasphaera micronuciformis ↑	0.3722	0.425705	-0.7616	0.006563	0.013126	-2.80311
Capnocytophaga gingivalis ↑	0.383134	0.425705	0.611173	0.758605	0.842895	-0.31388
Prevotella oris ↑*	0	0	1.415871	0.317877	0.45411	1.000628
Mycobacterium tuberculosis ↑*	0	0	1.837926	0.144178	0.240296	1.721885
Alloprevotella rava ↑	0.345267	0.425705	-0.72013	0.001289	0.006444	-2.65588

Supplementary Figures

a

	314 samples (after filtering)			48 samples selected using PSM		
	**All**	**TB**	**TBCOVID**	**All**	**TB**	**TBCOVID**
**Male**	236 (75.4%)	213 (74.7%)	23 (82.1%)	41 (85.4%)	21 (87.5%)	20 (83.3%)
**Female**	77 (24.6%)	72 (25.3%)	5 (17.9%)	7 (14.6%)	3 (12.5%)	4 (16.7%)

b

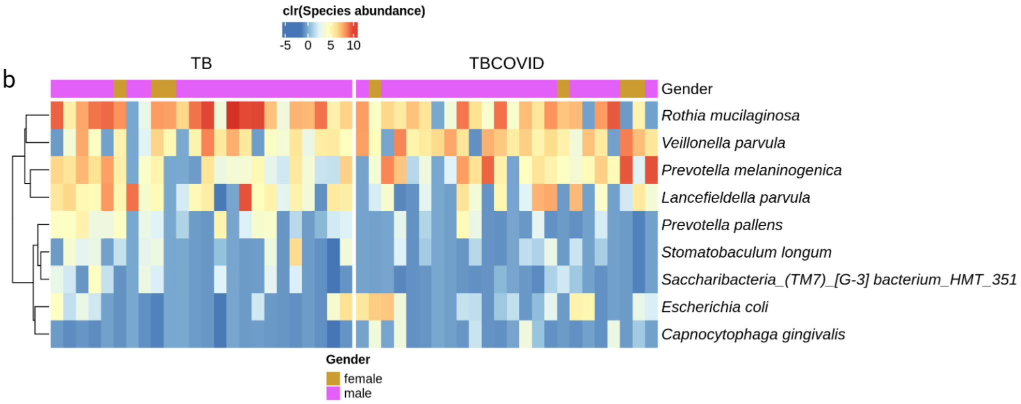

Fig. S1 **Significance of Gender covariate:** a) The distribution of males and females in the 314 samples used for matching, and in the 48 samples selected through PSM, is shown. In the full dataset before PSM, the ratio of males to females in the TB group differed from that in the TBCOVID group. However, after sample selection through PSM, the two groups showed similar males-to-females ratios. b) The heatmap shows species found to be differentially abundant between the TB and TB-COVID groups (see Fig. 5 in the main text for details). Gender is indicated in the top annotation bar.

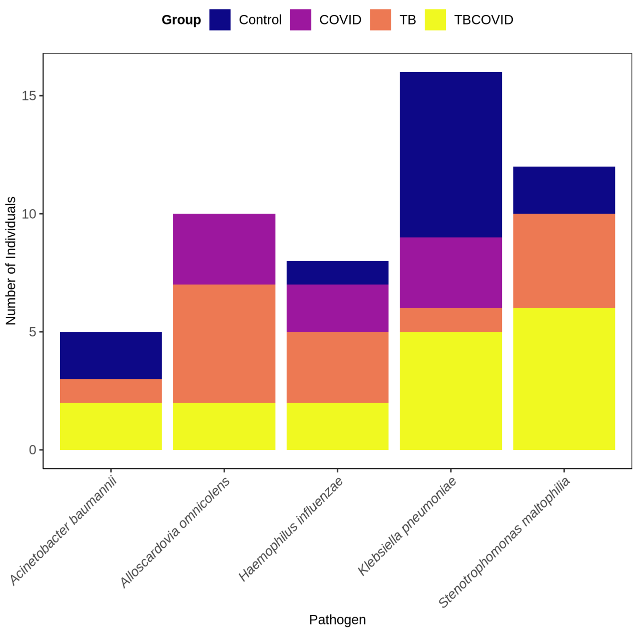

Fig. S2 **Pathogen analysis:** Distribution of respiratory pathogens across the four groups.

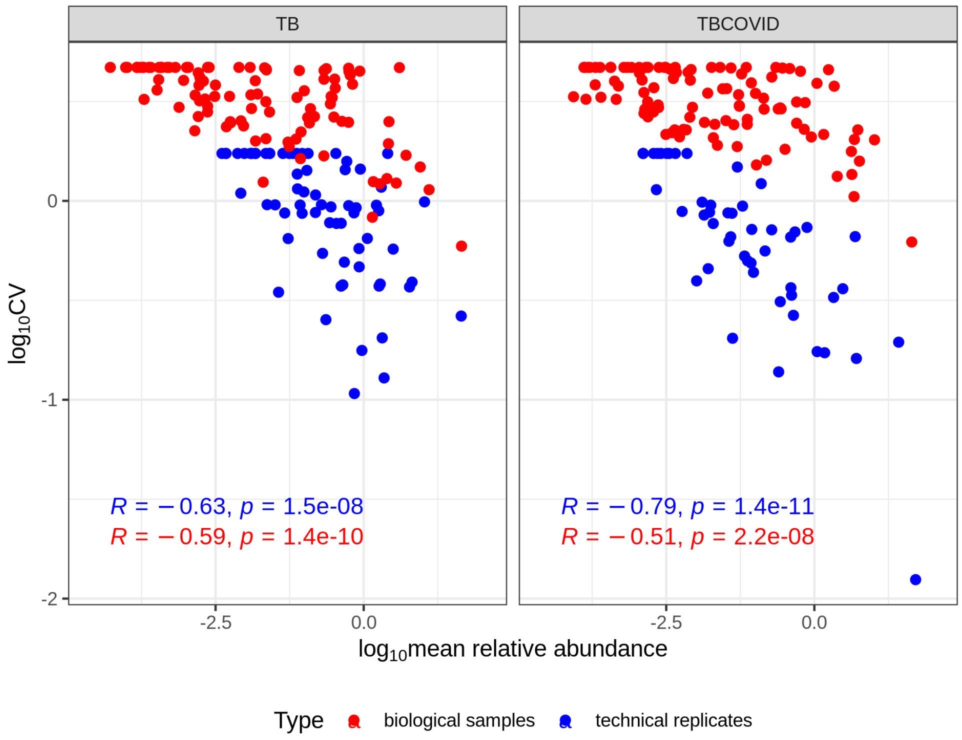

Fig. S3 **CV versus genus abundance across biological samples and technical replicates:** A sample with three technical replicates was used to assess technical variation. Samples without technical replicates were used to evaluate biological variation. Each point in the plot represents a genera.

To expand a bit more, to assess technical variation, we subjected a TB sample to three repeat measurements – the CV and mean of the relative abundance of each genera across these three technical replicates is shown as a blue dot in the left panel. Similar plot is shown for a TBCOVID sample with three technical replicates in the right panel.

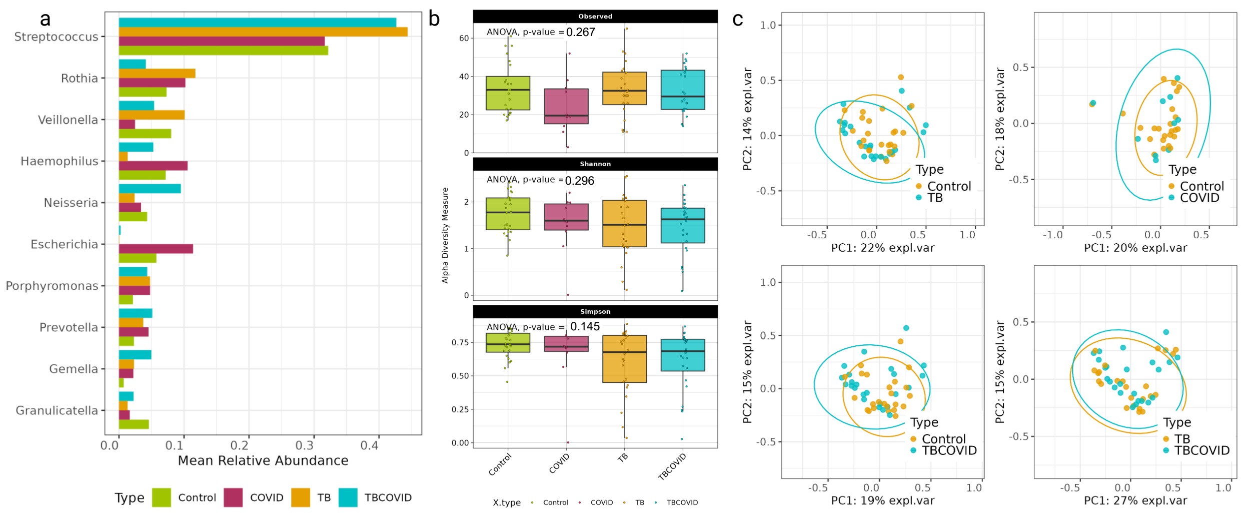

Fig. S4 **Characterization of genus diversity and composition in metagenomic sequencing data obtained from the four groups:** a) Visualization of the top 10 genera across the four groups, ranked based on their mean relative abundance. b) Boxplots of the alpha diversity measures (Observed, Shannon and Simpson) at genus level across the 4 groups. c) PCoA plots based on the Bray-curtis dissimilarity measure computed between the genus abundance profiles of samples.

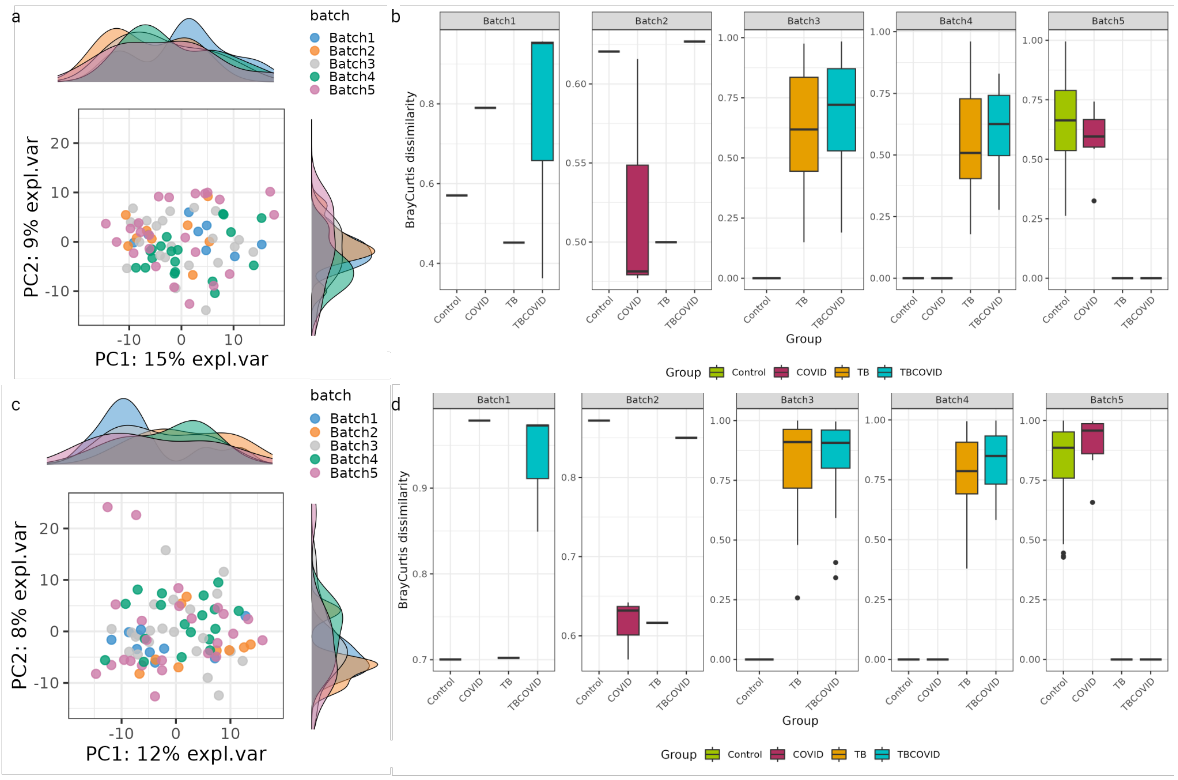

Fig. S5 **Batch effect analysis :** a) PCA plot of genus abundance across batches b) Bray-Curtis dissimilarity of samples within each group across batches based on genus abundance c) PCA plot of species abundance across batches d) Bray-Curtis dissimilarity of samples within each group across batches based on species abundance.

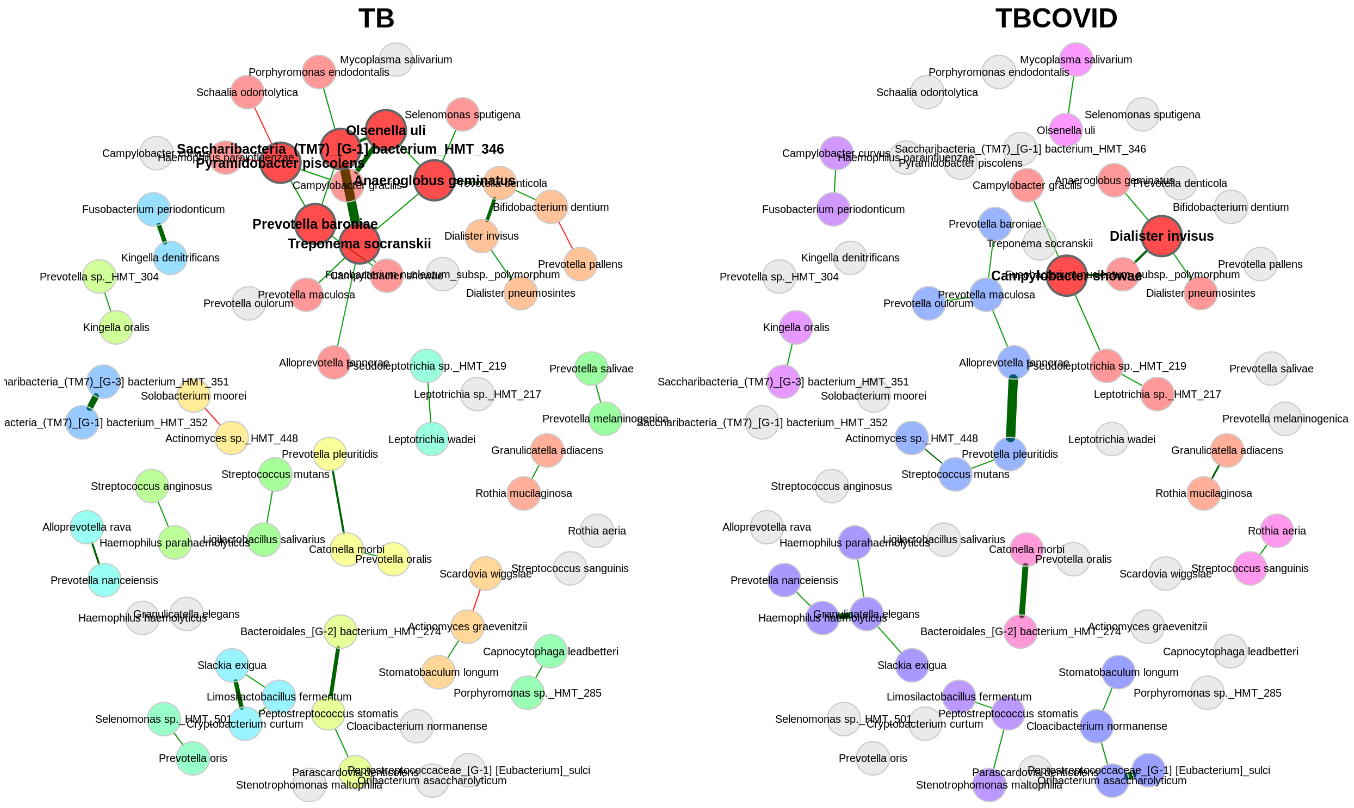

Fig. S6 **Network analysis results:** Association networks of TB and TBCOVID groups constructed using SpiecEasi method. The green and red colors of edges indicate positive and negative associations respectively. The thickness of edges corresponds to the strength of association, with thicker edges representing stronger associations. Only taxa present in at least 10% of samples within each group were used in the network construction.

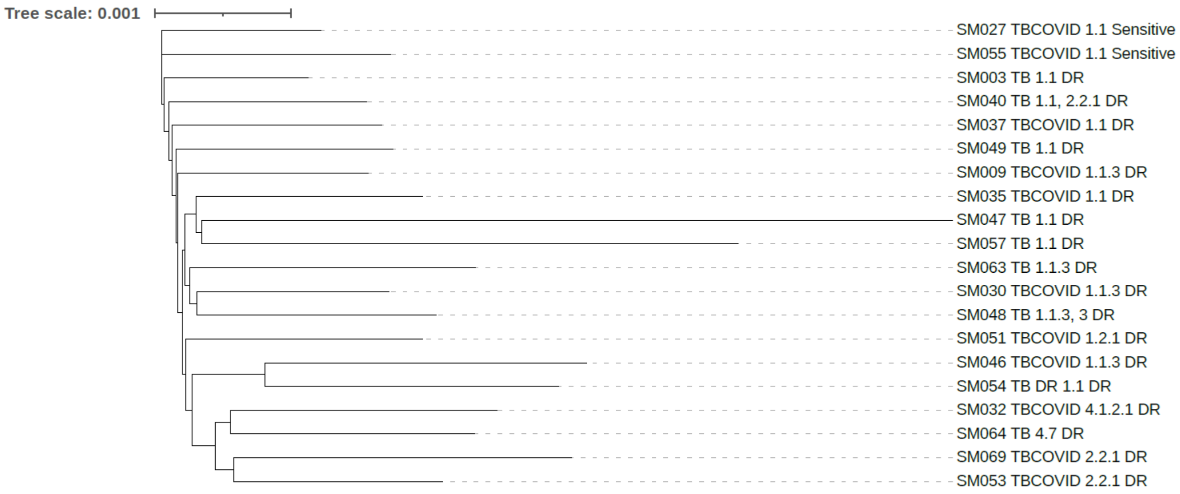

Fig. S7 **Genome analysis results:** Phylogenetic tree of the 9 TB and 11 TBCOVID samples constructed using the FastME/OneClick pipeline from the NGPhylogeny platform. The labels on the leaves indicate the group (TB/TBCOVID), lineages identified and drug resistance status (whether drug-resistant (DR) or sensitive). Two TB samples (SM040 and SM048) were identified as mixed infections containing two different strains of *M. tb*, with the major strain belonging to lineage 1.

Supplementary Files

Supplementary data/result files listed below are available at this link: https://drive.google.com/drive/folders/1o7sikxfRSLLFTl3VZUGB8rAAxy4tlANZ?usp=drive_link.

Suppl File D1a: The covariates of all 461 TB individuals from whom sputum specimens were obtained.

Suppl File D1b: Sample identifiers (IDs) and the associated covariates of samples selected for sequencing across the four groups including replicates.

Suppl File D2: This file contains the raw and post-filtering read counts for each sample.

Suppl File D3: This file contains abundance data at the **genus**, **species**, and **pathway** levels. Genus and species read counts were generated using custom R scripts, while pathway abundances were obtained from PICRUSt2 analysis.

Suppl File D4: This file contains results from **genus**-level DA analysis for different comparisons of two groups (chosen from the four groups: TB-only, COVID-only, TBCOVID and Controls). The results of each pairwise comparison is in a separate tab in this xlsx file, and include the p-values and adjusted p-values (q-value) obtained by the three methods corncob, ANCOM-BC and LinDA for each **genus**. Note that corncob does not give log fold change (LFC) and only the LFC's for ANCOM-BC and corncob are included.

Suppl File D5: This file contains results from **species**-level DA analysis for different comparisons of two groups (chosen from the four groups: TB-only, COVID-only, TBCOVID and Controls). The results of each pairwise comparison is in a separate tab in this xlsx file, and the p-values and adjusted p-values (q-value) obtained by the three methods corncob, ANCOM-BC and LinDA for each **species**. Note that corncob does not give LFC and only the LFC's for ANCOM-BC and corncob are included.

Suppl File D6: This file contains results from **pathway**-level DA analysis for different comparisons of two groups (chosen from the four groups: TB-only, COVID-only, TBCOVID and Controls). The results of each pairwise comparison is in a separate tab in this xlsx file,

and the p-values and adjusted p-values (q-value) obtained by the three methods corncob, ANCOM-BC and LinDA for each **pathway**. Note that corncob does not give LFC and only the LFC's for ANCOM-BC and corncob are included.

Suppl File D7: This file contains treatment status of patients in the TB and TBCOVID groups. Conditions such as drug resistance, multiple TB episodes, and death were combined into a single column labeled 'Adverse outcome'. The treatment status is extracted from the Nikshay portal during February 2025.
